# Muscle Fatigue in the Three Heads of Triceps Brachii During Intensity and Speed Variations of Triceps Push-Down Exercise

**DOI:** 10.3389/fphys.2020.00112

**Published:** 2020-02-21

**Authors:** Jawad Hussain, Kenneth Sundaraj, Indra Devi Subramaniam, Chee Kiang Lam

**Affiliations:** ^1^Centre for Telecommunication Research & Innovation, Fakulti Kejuruteraan Elektronik & Kejuruteraan Komputer, Universiti Teknikal Malaysia Melaka, Malacca, Malaysia; ^2^Centre for Technopreneurship Development, Pusat Bahasa & Pembangunan Insan, Universiti Teknikal Malaysia Melaka, Malacca, Malaysia; ^3^School of Mechatronic Engineering, Universiti Malaysia Perlis, Perlis, Malaysia

**Keywords:** triceps brachii, muscle fatigue, isotonic contractions, intensity variation, speed variation, sEMG

## Abstract

The objective of this study was to investigate the effects of changes in exercise intensity and speed on the three heads of the triceps brachii (TB) during triceps push-down exercise until task failure. Twenty-five subjects performed triceps push-down exercise at three different intensities (30, 45, and 60% 1RM) and speeds (slow, medium, and fast) until failure, and surface electromyography (sEMG) signals were recorded from the lateral, long and medial heads of the TB. The endurance time (ET), number of repetitions (NR) and rate of fatigue (ROF) were analyzed. Subsequently, the root-mean-square (RMS), mean power frequency (MPF) and median frequency (MDF) under no-fatigue (NF) and fatigue (Fa) conditions were statistically compared. The findings reveal that ROF increases with increase in the intensity and speed, and the opposite were obtained for the ET. The ROF in the three heads were comparable for all intensities and speeds. The ROF showed a significant difference (*P* < 0.05) among the three intensities and speeds for all heads. The three heads showed significantly different (*P* < 0.05) MPF and MDF values for all the performed exercises under both conditions, whereas the RMS values were significantly different only under Fa conditions. The current observations suggest that exercise intensity and speed affect the ROF while changes in intensity do not affect the MPF and MDF under Fa conditions. The behavior of the spectral parameters indicate that the three heads do not work in unison under any of the conditions. Changes in the speed of triceps push-down exercise affects the lateral and long heads, but changes in the exercise intensity affected the attributes of all heads to a greater extent.

## Introduction

Triceps brachii (TB) is the largest arm muscle responsible for elbow extension and horizontal arm abduction and also participates as an antagonist muscle during elbow flexion (Hussain et al., 2018). This muscle comprises three heads, namely, the long, lateral and medial heads. The long head, a bi-articular muscle, originates from the infraglenoid tuberosity of the scapula and participates during shoulder extension (Le Hanneur et al., 2018). The lateral and medial heads originate from the posterior surface of the humerus superior and inferior to the radial groove, respectively (O’Donnell et al., 2018). The lateral and long heads converge into one tendon that inserts into the olecranon, whereas the medial head is attached to the olecranon through a deeper and initially separated tendon (Madsen et al., 2006).

Landin et al. (2018) reviewed the functionality of TB in humans and noted that the medial head is involved in all types of elbow extensions, whereas the lateral and long heads participate in elbow extension against some resistance. The medial head is fully involved in elbow extension when the elbow is flexed beyond 90° (Madsen et al., 2006). As observed in a previous study (Murray et al., 2000), the long head maintains a relatively constant force-generating capacity during isometric contractions over different elbow angles. Additionally, the bi-articular nature of the long head (Davidson and Rice, 2010) causes different activation levels at different shoulder extension angles. The structure of each head suggests that these have distinct functionalities that might be observed through surface electromyography (sEMG).

The application of sEMG for assessing the TB during various activities was previously reviewed by Ali et al. (2014) and Hussain et al. (2018), and these reviews found that the majority of findings were centered around a single head. Some recent studies investigated the three heads separately and simultaneously (Davidson and Rice, 2010; Landin and Thompson, 2011; Ali et al., 2013; Kholinne et al., 2018; Hussain et al., 2019) during isometric contractions and concluded that the three heads do not work in unison. Madsen et al. (2006) performed an anatomical study of the TB throughout the elbow extension maneuver and drew a similar conclusion regarding the three heads. Two previous investigation (Ali et al., 2016; Hussain et al., 2020) studied the three heads during cricket bowling and triceps push-down exercise and appears to be the only instances when the three heads of the TB were observed separately during dynamic contractions. To the best of our knowledge, the three heads of the TB have not been previously observed, neither separately nor simultaneously, during both isometric and isotonic (dynamic) elbow extension maneuvers against resistance.

Isotonic movements, which are movements in which a muscle contracts and relaxes against a constant load, are believed to build muscle mass, endurance and muscle strength faster than isometric and isokinetic exercises (McArdle et al., 2015; Steele et al., 2017). The observation of muscle activity throughout the complete range of motion (ROM) of isotonic contractions is interesting because these movements are produced against a constant inertial load. The triceps push-down exercise is an isotonic exercise that involves the full ROM of elbow extensors against a load. The physiological attributes of the muscles may vary by manipulating the exercise variables, such as exercise intensity and speed. The gain in strength and the manifestation of fatigue in muscles varies with exercise intensity (de Salles et al., 2009). Also, variations in intensity induce changes in neural adaptations and hence the attributes of a muscle (Sale et al., 1983). Change in the exercise speed alters some important factors, such as time under tension, training volume, strength development and metabolic response of a muscle (Pereira et al., 2016; Wilk et al., 2018). During exercise at slow speeds, muscles remain under tension for a longer time, which is beneficial for strength gain (Burd et al., 2012), whereas fast speeds induce impulsive changes that are not long lasting (de Salles et al., 2009).

Peripheral muscle fatigue (hereafter fatigue) can be defined as a decrease in the capability of a muscle or group of muscles to produce force during or after a task (Bigland-Ritchie and Woods, 1984). A previous study (Selen et al., 2007) found that loss in the force-generating capacity of individual motor units (MUs) causes fatigue, and to overcome fatigue, the central nervous system attempts to increase the drive that causes the already recruited MUs to fire more rapidly and/or recruit new MUs. As fatigue progresses, the number of active MUs decreases, the CV of muscle fibers decreases (Buchthal et al., 1955; Stalberg, 1966), and the firing rate of MUs slows. These effects lead to the synchronization of MUs (Arihara and Sakamoto, 1999), which causes a decrease in the median (or mean) frequency of sEMG signals and an increase in the root mean square (RMS) amplitude, and persistence of these effects leads to eventual failure (Merletti et al., 1990).

Surface electromyography has been widely used to assess muscle activation during isotonic exercises (Zemková and Hamar, 2018; Latella et al., 2019). As detailed in the literature, a variety of parameters have been used for the assessment of muscle activity. The endurance time (ET) and number of repetitions (NR) in an exercise play a key role in strength training (Ammar et al., 2018; Malmir et al., 2019). The RMS of sEMG signals is considered an important indicator of muscle activation and has been used by numerous researchers (Christie et al., 2009; Sakamoto and Sinclair, 2012). In addition to the aforementioned temporal parameters, many researchers (Combes et al., 2018; Whittaker et al., 2019) have used the mean power frequency (MPF) and median frequency (MDF) of sEMG signals to analyze muscle fatigue. Spectral parameters such as MPF and MDF tend to decrease with the advent of muscle fatigue, and their rates of decrease are termed rate of fatigue (ROF) (Gerdle and Fugl-Meyer, 1992; Cifrek et al., 2009; Yung et al., 2012; Cruz-Montecinos et al., 2018), which is frequently used to analyze the fatiguing effects of exercise on muscles. Temporal parameters are more affected by external factors, such as the ROM (Sella, 2000), exercise type and intensity (Yung et al., 2012), muscles involved and equipment used, whereas spectral parameters appear to be independent of the exercise intensity and speed (Sakamoto and Sinclair, 2012).

The aim of this work was to investigate the effect of changes in exercise intensity and speed on each head of the TB during isotonic contractions under both no-fatigue (NF) and fatigue (Fa) conditions. Fatigue is an important phenomenon that restricts the efficiency of a muscle to perform a specific task, and the analysis of the three heads of the TB during fatiguing conditions is thus important. It has been hypothesized that fatigue impacts each of the three TB heads differently, and this hypothesis was tested using different exercise intensities and speeds. ET and NR were used to compare the effects of changes in the intensity and speed of exercise on the TB in general and the ROF of each head in particular. In addition, the RMS, MPF, and MDF of the sEMG signals from the three heads of the TB were utilized to examine the variations in the attributes of the three heads under NF and Fa conditions.

## Materials and Methods

### Participants

Twenty-five healthy, untrained, active, male university students participated in this study. The recruited subjects had no history or ongoing diagnosis of upper body neuromuscular disorder. The age, height and weight of the subjects were 23.8(3.6) years, 169.1(5.5) cm and 71.2(11.2) kg, respectively. The experimental protocol was approved by the Medical Research and Ethics Committee of Malaysia and adhere to the recommendations established by the Declaration of Helsinki. Instructions were given to the subjects prior to the experiment, and written informed consent was obtained. The experiment was performed at the university gymnasium, and a physician was available to aid the researchers and handle any emergency.

### Experimental Set-Up

**FIGURE 1:**
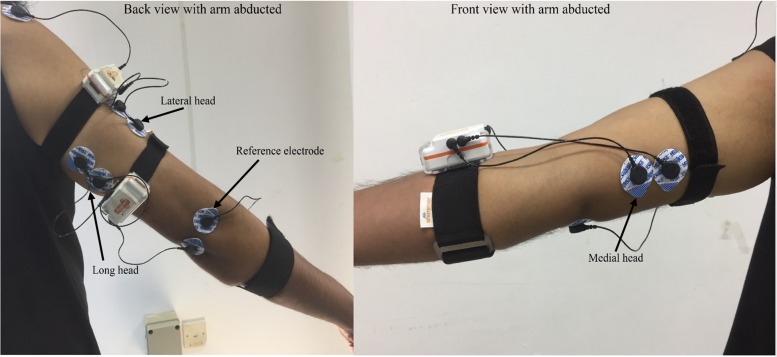
Placement of electrode over the lateral, long and medial heads of TB.

The three heads of the TB were observed using disposable pre-gelled bipolar sEMG electrodes (Kendall^TM^ 100 MediTrace^®^, Tyco Healthcare Group, United States). The heads were identified with the aid of a physician as described by Perotto (2011), and based on SENIAM’s recommendations, the electrodes were placed on the belly of each head in line with the muscle fibers. For electrode placement, a straight line was considered between the posterior crista of the acromion and the olecranon. The electrodes for the lateral and long heads were placed two finger-widths lateral and medial to the midpoint of the line, respectively. The electrodes for the medial head were placed at a distance of 4 cm proximal to the medial epicondyle of the humerus. The reference electrodes were placed over the lateral epicondyle and olecranon of the shoulder and elbow, respectively. Placement of electrodes is shown in Figure 1. The inter-electrode distance was 20 mm, and the skin was shaved, abraded and cleaned prior to electrode placement.

The sEMG signals were recorded using Shimmer 2.0r Model SH-SHIM-KIT-004 (Realtime Technologies Ltd., Ireland) with a frequency range of 5–322 Hz, a gain of 640, a common mode rejection ratio of 80 dB and a 12-bit ADC output. This wireless system consisted of three strap-on three-channel shimmer boards, each with dimensions of 53 mm × 32 mm × 15 mm and weighing approximately 25 g. Each Shimmer board was connected to one of the heads of the TB. The system was interfaced with a computer via class 2 Bluetooth^®^. The raw sEMG signals were recorded at a sampling rate of 1 kHz, as recommended by the manufacturer. The computer was placed at a distance of 2 to 3 m from the subject, and a line of sight was maintained between the computer and subject. The Shimmer Sensing LabVIEW program provided with the device was used to store the obtained data on the computer.

### Experimental Procedure

The electrodes were placed on the dominant arm of the subject prior to the familiarization session. The subjects were then asked to warm up, and the warm-up session consisted of upper body stretching and triceps push-down exercise with eight to ten repetitions using the lowest weights provided by the triceps push-down machine. The subjects were then given a resting period of approximately 2 min.

**FIGURE 2:**
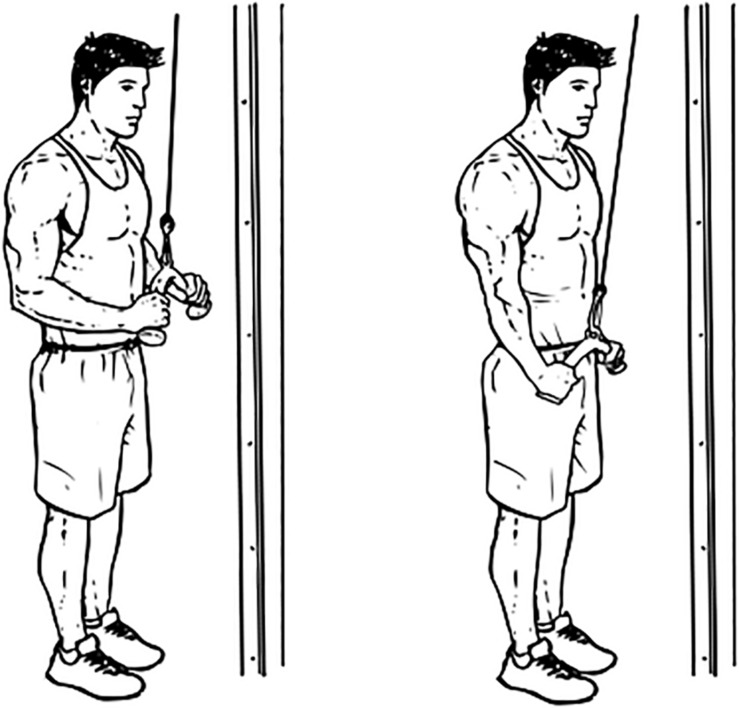
Triceps push-down exercise (source: www.workoutlabs.com).

The subject then stood in front of the triceps push-down machine and held the straight-bar with both hands in the pronated position at shoulder width. The subject maintained his arms close to, but not touching, the body and perpendicular to the ground with his torso slightly leaned forward to ensure that the bar did not touch the body during full extension. The subject moved his forearm toward the ground while maintaining the above-described posture until full elbow extension and then returned it to the starting position; this movement was considered one repetition of the full ROM. The correct posture was maintained throughout the ROM, as monitored by an assistant present on site, and the assistant also ensured that the subject did not use his body weight to move the bar. The maximal load that each subject held while performing one repetition successfully was termed “1 repetition maximum” (1RM). The subject was allowed an inter-exercise rest period of at least 15 min after determination of the 1RM. Figure 2 demonstrate the triceps push-down exercise.

Following the 1RM test in the familiarization session, the subject was asked to perform submaximal TB push-down exercise. The submaximal exercises were divided into three sessions that were separated by an inter-session rest of at least 24 h, and each session included an inter-exercise rest period of at least 15 min. The exercises were randomly assigned to each subject upon his arrival at the experiment site. The subject performed submaximal triceps push-down exercise at three different intensities (30, 45, and 60% 1RM) and maintained a tempo selected by the subject. The subject also performed the exercise with a 45% 1RM load at three different speeds, namely, slow, medium, and fast, and these speeds were controlled by a metronome. After some pilot testing, the metronome tempo was set to 80 and 120 beats per minute for the slow and medium speeds, respectively. Each repetition consisted of five beats, and the tempo was set to 3 beats down (concentric) and 2 beats up (eccentric). These tempos for the slow and medium speeds were approximately equivalent to 3.75 s and 2.5 s per repetition, respectively. For the fast condition, the subjects were asked to perform the exercise at the maximal possible speed while maintaining proper posture.

A custom-made program in LabVIEW measured the duration of a complete repetition from the real-time sEMG data, and it was ensured that all the repetitions were within ± 15% of this duration. No pause was allowed during contraction transition (from eccentric to concentric or vice-versa). Each participant performed the exercise until exhaustion, and the exercise was terminated if the subject could not control the speed of the bar during the eccentric phase or maintain balance between his dominant and non-dominant arms during two consecutive repetitions. During the experiment, the subjects were continuously given verbal encouragement to exert maximal effort and maintain the tempo. If a subject was frequently unable to maintain the correct posture (i.e., his torso leaned too much or became straight, or his arm abduction varied), a new subject was recruited as a replacement.

### Data Analysis

**FIGURE 3:**
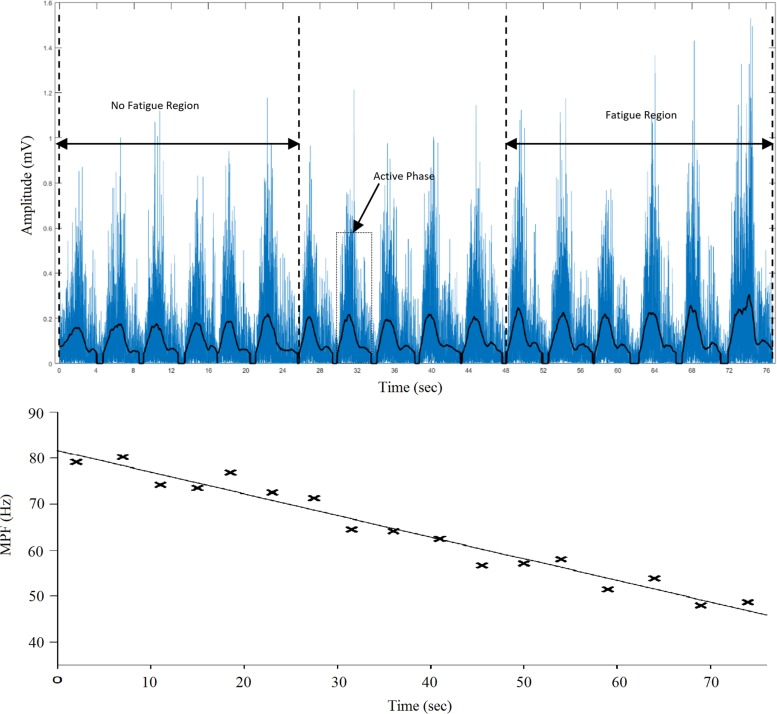
**(Top)**. Filtered and rectified sEMG signal from the lateral head of the TB of a subject from the start of exercise to task failure. The active phase and the NF and Fa regions are shown; **(Bottom)**. Best-fit line (slope = ROF) for the MPF of each active segment obtained through linear regression.

The sEMG data were recorded while the task was performed at 1RM and throughout the duration of the six exercises. The collected data (seven sEMG signals per subject – one for 1RM and six for the different exercises) were stored in a computer for further analysis. Custom-written programs in MATLAB 17 (MathWorks Inc., United States) were used to filter, normalize and evaluate the RMS, MPF, and MDF. A fourth-order bandpass Butterworth filter (cut-off frequencies of 5–450 Hz) was used to filter the data. A 512-point Short-Time Fourier Transform (STFT) computed with a 50% window overlap was used to estimate the MPF and MDF because the sEMG signals obtained during dynamic contractions are not stationary (Karlsson et al., 2008). The filtered and rectified sEMG signals obtained for each subject during each exercise were used to extract segments corresponding to the active phase (concentric and eccentric). A previous study (Rainoldi et al., 2000) revealed that the relative position of muscle fibers and the geometrical position of the sEMG electrodes over the muscles might vary during dynamic contractions. Because the placement of electrodes might alter the deductions or interpretations from the observed sEMG signals (Ahamed et al., 2012), the sEMG parameters associated with dynamic contractions might be calculated over the entire active phase, and a single value might represent the entire repetition. Although this method might not provide sufficient information on MU recruitment and firing rates, it can still be employed to deduce information regarding the development of fatigue in muscles. The active phases were identified using a 256-ms moving window to obtain the mean value for the signal with a threshold set to 15% of the maximal value from the entire recording, as shown in Figure 3. The RMS, MPF, and MDF were calculated for each active phase. Subsequently, the RMS was normalized against a dynamic contraction rather than a maximal voluntary contraction by considering the average RMS amplitude from the 1RM exercise. This approach was used due to the difficulty in finding the optimal joint angle that provides maximal output effort by all three heads. A similar approach was also used in a previous study (Sakamoto and Sinclair, 2012). The ET, which was defined as the time from the start of the exercise until task failure, and NR, which was defined as the number of active segments, were compared. For all three heads, the first and last six segments (NF and Fa, respectively) were identified (Figure 3), and the MPF, MDF, and normalized RMS were calculated for all the identified segments. The ROF was calculated from the slope of the MPF through regression analysis, as suggested in previous studies (Gerdle and Fugl-Meyer, 1992; Cifrek et al., 2009; Yung et al., 2012; Cruz-Montecinos et al., 2018).

### Statistical Analysis

For each subject, the ROF, ET, and NR of the three heads were obtained at different intensities and speeds, and the RMS, MPF, and MDF values were then obtained from the active phase under no-fatigue (NF) and fatigue (Fa) conditions. All the data were tested for normality using the Shapiro-Wilks test and were found to be normally distributed. One-way repeated-measures ANOVA was employed to perform the following comparisons: (1) ET and NR between three intensities (30, 45, and 60% 1RM) and between three speeds (slow, medium, and fast), (2) ROF between the three heads in the exercises performed at different intensities and different speeds, and (3) the RMS, MPF, and MDF of the three heads during exercise at different intensities and speeds under both conditions. Three-way repeated-measures ANOVA was used to study the main effects of (1) the exercise condition (NF and Fa), heads (lateral, long, and medial) and intensity (30, 45, and 60% 1RM) and (2) the exercise condition, heads and speed (slow, medium and fast). Finally, two-way repeated-measures ANOVA was used to observe the main effects of (1) the condition-intensity and (2) the condition-speed interactions on each head of the TB. Greenhouse-Geisser corrections were used for cases that violated the sphericity assumption, and Bonferroni adjustments were applied for the *post hoc* analysis. A selected dataset was considered significant if *P* < 0.05. IBM SPSS 20.0 (SPSS Inc., United States) was used for the statistical analyses.

## Results

**FIGURE 4:**
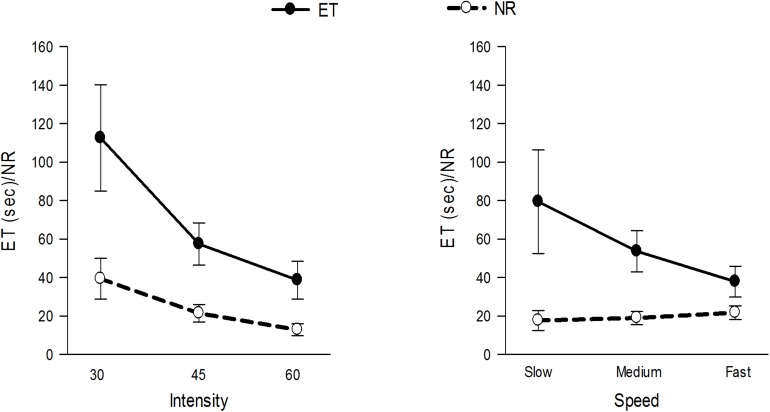
μ(SD) of the ET and NR at 30, 45, and 60% 1RM and at slow, medium and fast speeds.

**FIGURE 5:**
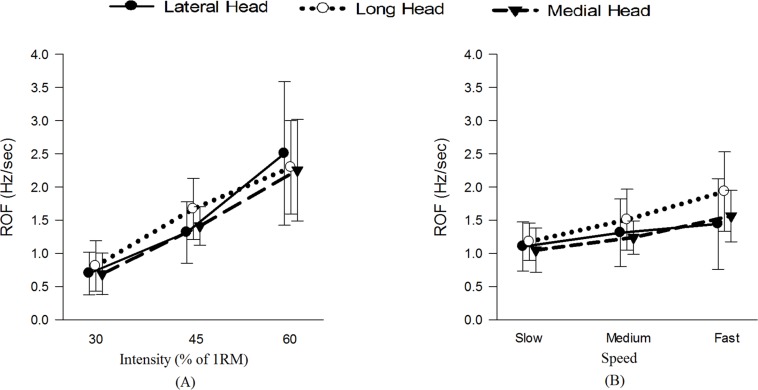
μ(SD) of the ROF in the three heads of the TB **(A)** at 30, 45, and 60% 1RM and **(B)** at slow, medium and fast speeds.

Figure 4 and Table 1 show and summarizes the μ(SD) values of the ET and NR for the observed exercise intensities and speeds. The ET decreased with increases in the exercise intensity and speed, and the NR also decreased with increases in the intensity but was not affected by the exercise speed. Figure 5 shows the μ(SD) values of the ROF in the three heads during different exercises, and as shown, the ROF exhibited an increasing trend with increases in both the intensity and speed of exercise.

**TABLE 1:** μ(SD) of the ET, NR, and ROF at different intensities and speeds.

Parameter	Intensity			Speed		
	30%	45%	60%	Slow	Medium	Fast
ET (sec)	112.59 (27.61)	57.43 (10.89)	38.66 (9.83)	79.45 (26.92)	53.66 (10.68)	37.86 (8.06)
NR	39.35 (10.62)	21.43 (4.54)	12.86 (3.11)	17.64 (5.22)	19.00 (3.44)	21.71 (3.56)
ROF_lat_ (Hz/sec)	0.70 (0.32)	1.31 (0.46)	2.50 (1.08)	1.10 (0.37)	1.31 (0.51)	1.44 (0.68)
ROF_lo_ (Hz/sec)	0.81 (0.38)	1.67 (0.46)	2.29 (0.70)	1.17 (0.28)	1.51 (0.46)	1.93 (0.60)
ROF_med_ (Hz/sec)	0.69 (0.31)	1.41 (0.29)	2.25 (0.77)	1.05 (0.33)	1.23 (0.25)	1.56 (0.39)

Table 2 summarizes the statistical comparisons of the ROF under different combinations. The three heads showed significant differences during the triceps push-down exercise at fast speed. All heads of the TB showed significantly different intensities (*P* < 0.05) for ROF, and the *post hoc* analysis revealed significant differences in all the heads among all intensity pairs with the exception of the long head between 45 and 60% 1RM. Among the speeds, only the long and medial heads showed significant differences (*P* < 0.05).

**TABLE 2:** Results of one-way repeated-measures ANOVA of the ROF (*P* value) and *post hoc* tests.

Comparison of the three heads					
**30%**	**45%**	**60%**	**Slow**	**Medium**	**Fast**

0.155	0.113	0.471	0.720	0.151	**0.005^a,b^**

**Comparison of the three intensities**			**Comparison of the three speeds**		
**Lateral**	**Long**	**Medial**	**Lateral**	**Long**	**Medial**

**<0.001^d,e,f^**	**<0.001^d,f^**	**<0.001^d,e,f^**	0.096	**<0.001^g,i^**	**0.031^i^**

*Bold font indicates statistical significance (*a* – lateral and long, *b* – long and medial, *c* – lateral and medial, *d* – 30% and 45%, *e* – 45% and 60%, *f* – 30% and 60%, *g* – slow and medium, *h* – medium and fast, *i* – slow and fast).

**FIGURE 6:**
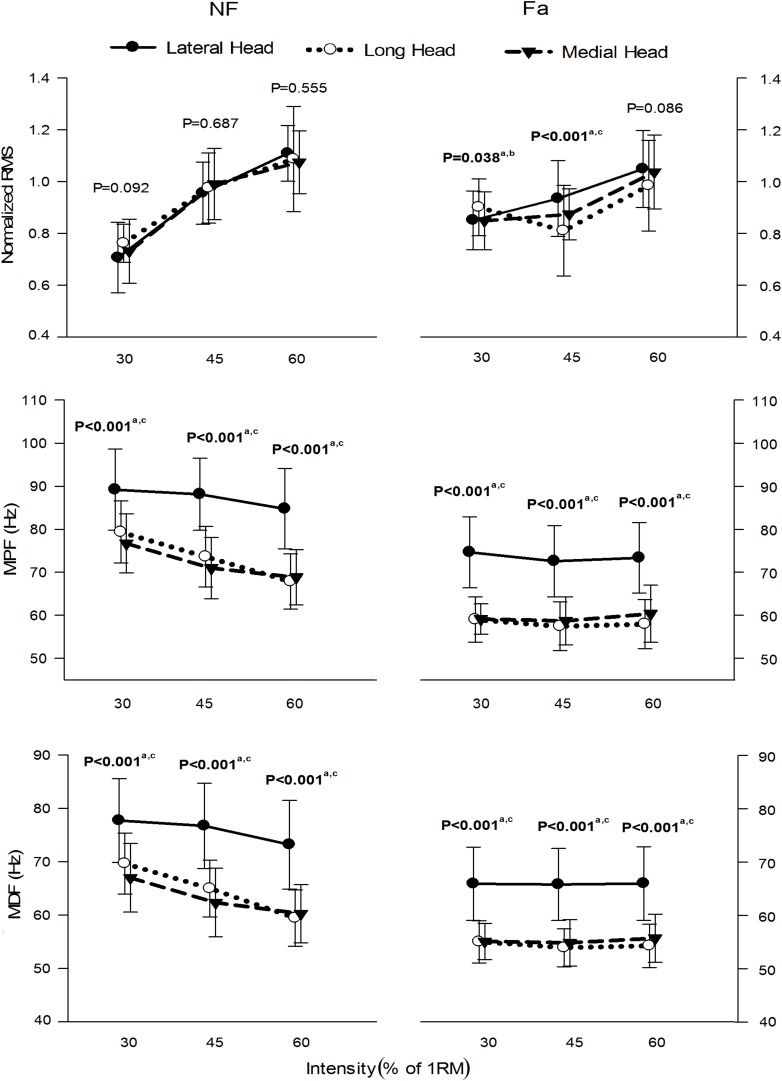
μ(SD) of the normalized RMS, MPF, and MDF of the three heads of the TB at 30, 45, and 60% 1RM under NF and Fa conditions. Bold font indicates statistical significance (a – lateral and long, b – long and medial, c – lateral and medial).

Figure 6 presents the normalized RMS amplitude, MPF and MDF of the three heads of the TB during the whole active phase at different intensities under NF and Fa conditions. The three heads showed significant differences (*P* < 0.05) in the RMS, MPF, and MDF among all intensities under both NF and Fa conditions with the exception of the RMS under NF conditions. The three-way ANOVA results revealed that all main effects and interactions were statistically significant (*P* < 0.05) for all the observed parameters (Table 3). The two-way ANOVA results revealed that the condition × intensity interaction was significant only for the RMS, MPF, and MDF of the long and medial heads (*P* < 0.05). For all observed parameters, the main effect of the exercise intensity was only significant in the long head (*P* < 0.001), whereas the main effect of the exercise condition was statistically significant in all three heads (*P* < 0.001). All three heads demonstrated a decrease in amplitude from NF to Fa conditions at higher intensities (45 and 60%), and the long head exhibited the greatest decrease. The lateral head exhibited the highest MPF and MDF under both NF and Fa conditions (*P* < 0.05) and showed the greatest decrease in the MPF and MDF from the NF to Fa conditions.

**TABLE 3:** *P* value for the main effects of the fatiguing conditions, TB heads, intensity levels and their interactions on the different parameters (*n* = 25).

Effect	Parameter		
	RMS	MPF	MDF
Condition	**> 0.001**	**> 0.001**	**> 0.001**
Head	**0.021**	**> 0.001**	**> 0.001**
Intensity	**> 0.001**	**0.010**	**0.048**
Condition × Head	**0.003**	**> 0.001**	**> 0.001**
Condition × Intensity	**> 0.001**	**> 0.001**	**> 0.001**
Head × Intensity	**0.030**	**0.001**	**> 0.001**
Condition × Head × Intensity	**> 0.001**	**> 0.001**	**0.015**

*Bold font indicates statistical significance.

**FIGURE 7:**
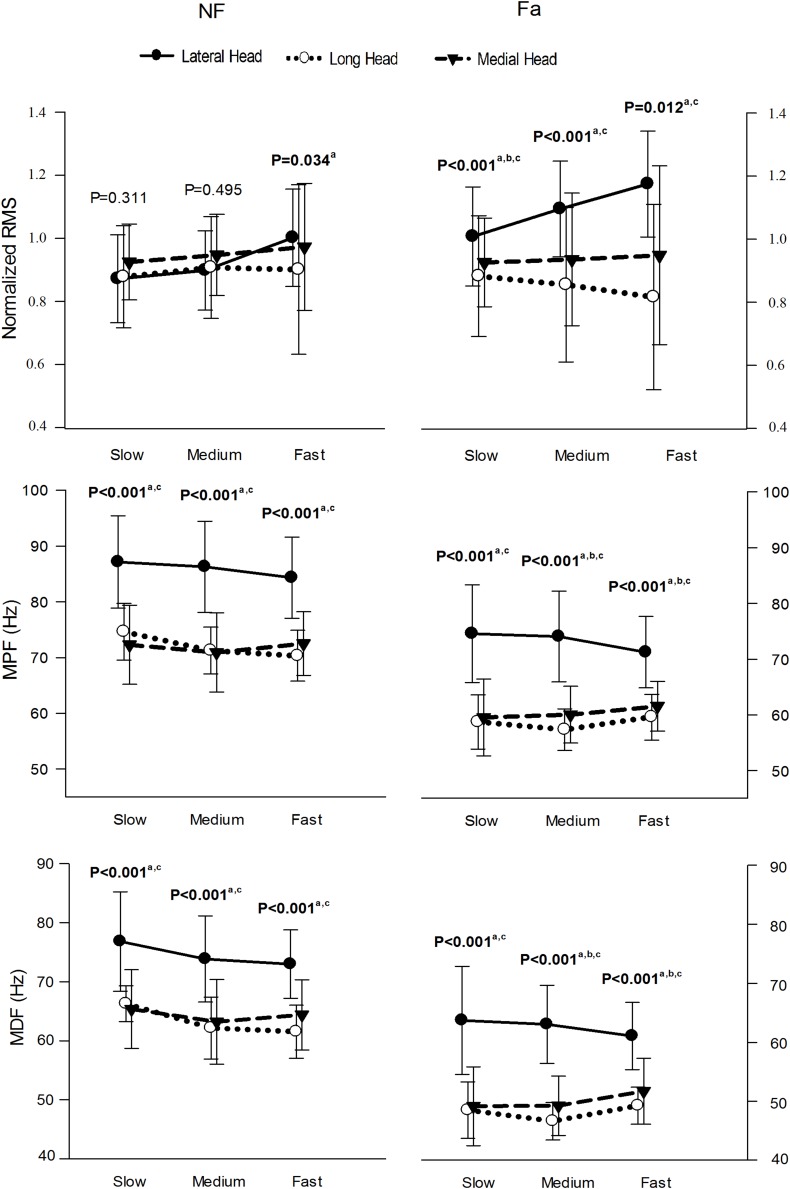
μ(SD) of the normalized RMS, MPF, and MDF of the three heads of the TB at slow, medium and fast speed in NF and Fa condition. Bold font indicates statistical significance (a – lateral and long, b – long and medial, c – lateral and medial).

Figure 7 presents the normalized RMS amplitude, MPF and MDF of the three heads of the TB during the whole active phase at different speeds under NF and Fa conditions. The three heads showed significant differences (*P* < 0.05) in the RMS, MPF, and MDF at all the speeds under both NF and Fa conditions with the exception of the RMS under NF conditions. The three-way ANOVA results revealed that all main effects and interactions with the exception of the condition × speed interaction were statistically significant (*P* < 0.05) for all the observed parameters (Table 4). The two-way ANOVA results revealed that the condition × speed interaction was only significant for the RMS, MPF, and MDF of the long head (*P* < 0.001). The main effect of speed was significant for all the parameters of the lateral head (*P* < 0.001) but only the spectral parameters (MPF and MDF) of the long head. The main effect of the exercise condition was significant for only the spectral parameters of all the heads (*P* < 0.001). The highest change in amplitude from NF to Fa conditions was observed in the lateral head, whereas the long head exhibited the highest decrease in the MPF and MDF at all observed speeds.

**TABLE 4:** *P* value for the main effects of the fatiguing conditions, TB heads, different speeds and their interactions on the different parameters (*n* = 25).

Effect	Parameters		
	RMS	MPF	MDF
Condition	**0.001**	**> 0.001**	**> 0.001**
Head	**0.025**	**> 0.001**	**> 0.001**
Speed	**> 0.001**	**0.002**	**> 0.001**
Condition × Head	**> 0.001**	**0.001**	**0.002**
Condition × Speed	0.111	0.068	0.054
Head × Speed	**> 0.001**	**> 0.001**	**> 0.001**
Condition × Head × Speed	**> 0.001**	**> 0.001**	**0.010**

*Bold font indicates statistically significant.

## Discussion

This study was undertaken to investigate the effects of changes in the intensity and speed of triceps push-down exercise on the three heads of the TB. Specifically, the study investigated the hypotheses that changes in exercise intensity and speed affect fatigue, and that their effects on the three heads are different. For this, the ET and NR for exercises at different intensities and speeds were observed. The ROF was compared at the three heads. Subsequently, the RMS, MPF, and MDF were compared between the three heads under both NF and Fa conditions.

We found that the ROF increased with increases in the exercise intensity (Table 1 and Figure 5A). Because the exercise intensity significantly affects the physiological attributes of a muscle, an increase in the exercise intensity causes the muscle(s) to recruit more MUs and/or fire them more frequently (Xu et al., 2018), and these effects might induce greater decreases in the spectral parameters over time. The current study also revealed that the three intensities resulted in significantly different values of the ROF for all three heads of the TB. The long and medial heads exhibited the highest and lowest mean ROF values, respectively.

We observed the ET and NR to be higher at lower intensities (Figure 4), and concurs with the results of a previous study (Hsu et al., 2011). The higher ROF might be one of the important reasons explaining the lower ET and NR values obtained at higher intensities. Furthermore, because the TB is mainly composed of type II muscle fibers, which are more utilized during high-power impulsive activities (Johnson et al., 1973), these types of skeletal muscles are expected to fatigue early (Merletti et al., 2016), explaining the observed lower ET and NR values at higher intensities.

Higher intensities require greater MU recruitment, increased utilization of fast-twitch fibers and higher force production (Hammond et al., 2019), which explains the increase in the RMS amplitude obtained with higher intensities (Figure 6).

Our results demonstrate that the MPF and MDF of all the heads tend to decrease with increases in the exercise intensity under NF conditions, whereas these parameters exhibited no significant differences between different exercise intensities under Fa conditions (Figure 6). The reason for this behavior could be explained as follows. Besides other parameters such as size and type of muscle fibers and type of exercise, the net lactate concentration in muscles depends on the force (contraction) level. Higher contraction levels affect the blood flow in the muscles, thus effecting the removal of metabolic wastes. As this waste accumulates in the muscle over time, it causes a change in intracellular pH, thus decreasing the muscle fiber CV and causing the power spectrum to shift to lower frequencies. Persistence of this phenomenon causes peripheral fatigue in muscles. The MPF and MDF, that are related to CV, may vary at different effort levels during NF condition due to the difference in the utilization of muscle fibers and chemical state of muscles. During Fa condition, the fast twitch muscle fibers are switched-off and MUs activity become time synchronized which may cause similar spectral output. Also, each muscle attempts to optimize its energy consumption (Hales and Johnson, 2019) and MU recruitment under NF conditions, while the major focus during Fa is task performance rather than energy optimization. Thus, the trend of the spectral parameters is expected to differ among different exercise intensities for the two conditions. Yet another possible reason for this difference in the behaviors of these parameters under both conditions might be the different levels of participation by elbow flexors during co-contractions, suggesting the possibility of workload sharing among the three heads during elbow extension maneuvers under Fa conditions.

In addition to the exercise intensity, the exercise speed also impacts the attributes of a muscle. Previous researchers have demonstrated that performing exercise at higher frequencies results in a greater ROF (Hsu et al., 2011), and our results concur with this finding (Table 1 and Figure 5B). One of the possible reasons for this higher ROF might be restriction in the blood flow supply at higher speeds (Griffin et al., 2001), which might lead to insufficient oxygen delivery and inadequate removal of metabolic waste from muscles (Oyewole, 2014). The speed of exercise impacts the exercise volume (Wilk et al., 2018) with slower speeds allowing extension of exercise time, thus reducing ROF. Also, slow speed allows increased blood flow due to pumping effect of contracting muscles, thus handling the metabolic waste more efficiently. Furthermore, higher speeds require higher MU recruitment and firing rates that results in a higher ROF. It is interesting to note that the normalized RMS showed increasing trend with increase in exercise speed for lateral and medial heads only. The long head, being bi-articular demonstrated different activation patterns from other two heads at different joint angles (Kholinne et al., 2018). This could be a potential reason for long head demonstrating different behavior from the other two heads. No specific pattern was observed for MPF and MDF for the three speeds (Figure 7).

While, the ET was high at a slow-speed exercise, the NR at different speeds was comparable. This finding implies that although the muscles are fatigued at slower rates during slow-speed exercise, the work done by the muscle remains unchanged (Hellebrandt and Houtz, 1958; Moffroid and Whipple, 1970).

In the current study, the three heads demonstrated significantly different spectral behavior during triceps push-down exercise at different intensities and speeds under both NF and Fa conditions (Figures 6, 7). To the best of our knowledge, only two previous studies (Hussain et al., 2019, 2020) commented on the MPF and MDF of the three heads of the TB, and our results agrees with the findings. However, the *post hoc* analysis revealed an interesting observation. The long-medial head pair did not show significant differences between the intensities under both conditions. The behavior of this pair appears opposite to the other two synergist pairs (lateral-medial and lateral-long head pairs) which might be due to its different biomechanical structure. The long head is bi-articular, the lateral head is mono-articular, and both heads are of comparable size (Elder et al., 1982). In contrast, the medial head is mono-articular and smaller in size. The lateral-medial pair works in unison due to the mono-articular nature of these two heads, whereas the lateral-long pair works in unison due to the larger size of both these heads. The long-medial head pair, although being synergist, is the exception probably because it does not fit either of these two categories. Further, although the primary function of the medial head is elbow extension, it is only fully involved in the function when the elbow is extended from 0° to 90° (where 0° denotes full elbow extension) (Madsen et al., 2006). The long head, however, produces a relatively constant force generating capacity across a wider range of elbow angles (Murray et al., 2000), with comparatively higher activation levels at 0° shoulder elevation (Kholinne et al., 2018). Based on this, we could expect that the two heads would have similar activation patterns per intensity level during the TB push-down exercise.

Interestingly, although the three heads exhibited different spectral behavior, their ROF was comparable among the three heads (Figures 5A,B), and this finding is contradictory to our previous results (Hussain et al., 2019), which revealed that the ROF was statistically significant at lower intensities (30 and 45% MVC) during isometric elbow extensions. The reason for these contradicting results could be due to the different exercise types. Although phase transitions (from eccentric to concentric, etc.) does not involve any actual rest, they intrinsically allow muscles to relax (Hietanen, 1984), and this effect delays the onset of fatigue and increases the ET, thus affecting ROF. Further, as mentioned by Humphreys and Lind (1963), the blood flow that determines the rate of metabolic removal is usually restricted during isometric contractions, while the blood flow is increased during dynamic contractions due to pumping effect of muscles, hence different results might be expected for the two exercises.

The behavior of the normalized RMS of the three heads, however, was different from those of the MPF and MDF (Figures 6, 7). Under NF conditions, the normalized RMS of the three heads did not exhibit statistical significance, but statistical significance was observed under Fa conditions. The difference in the normalized RMS under both conditions indicates that the three heads function differently under NF and Fa conditions. As aforementioned, under NF conditions, the muscles tend to optimize their energy consumption (Hales and Johnson, 2019) and thus work independently, and these effects roughly lead to activation of the muscle to the exercise intensity level and hence result in the same normalized RMS values for all participating muscles. However, a different phenomenon is observed during fatigue because the key focus under these conditions is to perform the maneuver rather than energy optimization, and thus while the workload might not be distributed equally among muscles (Zhang et al., 1995), there is indication that workload sharing exists among the three heads during fatigue (Rojas-Martínez et al., 2019). While our results observe this to be true for synergist pairs, again the long-medial head pair observe comparable normalized RMS values which could be due to its peculiar biomechanical structure as aforementioned.

The comparison of NF and Fa conditions revealed that the spectral parameters of all the observed muscles showed significant differences for all the performed exercises. The RMS, however, showed significant differences only in the lateral head for the three speeds. These observations agree with the general findings detailed in the literature, which reports that the sEMG spectral parameters are better approximators of muscle fatigue than temporal parameters (Cifrek et al., 2009; González-Izal et al., 2012).

The approach used in this work could provide additional insights if the three heads were investigated during the eccentric and concentric phases separately. Such an approach would require the joint angles to be continuously monitored and synchronized with real-time sEMG data using additional sensors or motion capture system. Analyzing the sEMG signals from three heads at different joint angles during a triceps push-down exercise may further improve our understanding, on the individual biomechanical activation patterns and their combined compensation strategies, toward better insights on the role of TB during elbow extension. In addition, the inclusion of untrained subjects from only one gender and the fact that the exercises were performed neither at low intensities (less than 25% 1RM) nor at high intensities (greater than 70% 1RM) might probably limit the interpretation and generalization of findings observed in the current study. We also note that the since the agonist muscles were the only ones observed, the supporting role and contribution of the biceps brachii as an antagonist muscle during elbow extension cannot be entirely overruled.

## Conclusion

The results and observations obtained in this study reveal that the three heads of the TB work independently during triceps push-down exercise performed at different intensities and speeds. The ROF increased with increases in both the exercise intensity and speed, resulting in lower ET and NR values at higher intensities. For the tested exercise intensities, the MPF and MDF of all three heads tend to decrease with increases in the exercise intensity under NF conditions but remained the same under Fa conditions. Our findings also note that workload sharing among the three heads of the TB might occur during fatigue. Further analysis is required to quantify this workload sharing as well as the role of each head in a particular activity. Change in exercise intensity affect all the three heads of the TB but speed affects only the lateral and long heads. Changes in exercise speed do not affect the activation of a muscle but could influence the time under tension, and hence, trainers may focus more on the exercise intensity instead. The findings of the current study might aid the design of rehabilitation or targeted training programs for the individual heads of the TB, either for patients or athletes. In addition, our results may help roboticists and automation enthusiasts in the design and control of TB related prosthetics for the disabled.

## Data Availability Statement

The datasets for this study will not be made publicly available because the Medical Research and Ethics Committee (MREC) of Malaysia has imposed restrictions on making the underlying data of this study publicly available. The data may be provided upon request to the corresponding author or at airehab@utem.edu.my.

## Ethics Statement

The experiment protocol was approved by the Medical Research and Ethics Committee of Malaysia and was in line with Declaration of Helsinki. We gave instructions to the subjects prior to experiment and a written informed consent was taken. The experiment was performed at university gymnasium, and a physician was available to aid the researchers and handle any emergency.

## Author Contributions

JH and CL conceived and designed the search experiment. JH and CL performed the search experiment. JH, KS, and IS performed the contents arrangement. JH, KS, and IS wrote the manuscript.

## Conflict of Interest

The authors declare that the research was conducted in the absence of any commercial or financial relationships that could be construed as a potential conflict of interest.
